# A comparative analysis of CT angiography and echocardiography in the evaluation of chest findings in patients with interrupted aortic arch

**DOI:** 10.3389/fradi.2025.1616112

**Published:** 2025-06-26

**Authors:** Zhanar Moldakhanova, Raushan Rakhimzhanova, Tairkhan Dautov, Lazzat Bastarbekova, Bauyrzhan Kaliyev, Assel Almussina, Aizhan Zhankorazova, Nurmakhan Zholshybek

**Affiliations:** ^1^ Radiology Unit, Heart Center, University Medical Center, Astana, Kazakhstan; ^2^ Scientific Research Institute of Radiology Named After Zh.Kh. Khamzabayev, Astana Medical University, Astana, Kazakhstan; ^3^ Clinical and Academic Department of Radiology and Nuclear Medicine, University Medical Center, Astana, Kazakhstan; ^4^ Inpatient Radiology Unit, Mother and Child Center, University Medical Center, Astana, Kazakhstan; ^5^ Department of Medicine, School of Medicine, Nazarbayev University, Astana, Kazakhstan

**Keywords:** interrupted aortic arch, echocardiography, computed tomography angiography, congenital heart defects, diagnostic imaging, surgical planning

## Abstract

Interrupted aortic arch (IAA) is a rare congenital cardiovascular anomaly characterized by the absence of continuity between the ascending and descending aorta, often accompanied by congenital heart defects such as ventricular septal defects and patent ductus arteriosus. Accurate preoperative imaging is essential for surgical planning and patient management. This study aimed to compare the diagnostic accuracy of echocardiography and computed tomography angiography (CTA) in evaluating thoracic findings in patients with IAA. A retrospective analysis was conducted on 58 patients (median age: 18 days) diagnosed with IAA between September 2020 and January 2023 at the Heart Center, University Medical Center, Astana, Kazakhstan. Conventional echocardiography and multislice CTA were performed using standardized protocols. Sensitivity, specificity, and other diagnostic performance metrics were calculated. Statistical comparisons were made using McNemar's and Wilcoxon signed-rank tests, with *p* < 0.05 considered significant. Echocardiography correctly identified 91.4% of IAA cases, while CTA achieved 100% sensitivity and specificity. McNemar's test revealed a significant difference in diagnostic performance favoring CTA (*p* < 0.05). Measurements of the ascending aorta diameter showed no statistically significant difference between the two modalities (*p* = 0.09). IAA was predominantly type A (48.3%) and type B (46.6%), with hypoplastic ascending aorta identified in 34.5% of patients. Echocardiography remains a practical initial imaging modality for IAA, offering portability and cost-effectiveness. However, CTA demonstrated superior diagnostic accuracy and anatomical resolution, making it the preferred tool for detailed preoperative evaluation and surgical planning. Future studies with larger cohorts and additional modalities could further refine diagnostic strategies for IAA.

## 1 Introduction

Variants and anomalies of the aortic arch (AA) are relatively common. In its standard configuration, the AA is left-sided and gives rise to three branches: the right brachiocephalic artery, the left common carotid artery, and the left subclavian artery (1, 2). Among the various AA variants, the most frequent is the brachiocephalic trunk, where the brachiocephalic and left common carotid arteries share a common origin. Another notable variant is the direct aortic origin of the left vertebral artery. Common AA anomalies include a left-sided arch with an aberrant right subclavian artery, a right-sided arch, and a double AA (3).

Interrupted aortic arch (IAA) is regarded as one of the most severe forms of AA anomalies. While AA anomalies typically involve variations in the branching patterns or the anatomical positioning of the AA, IAA is a rare congenital cardiovascular malformation characterized by a complete luminal discontinuity of the AA, resulting in the absence of the regular aortic continuity between the ascending and descending aorta (4), distinguishing it from severe coarctation and aortic atresia (5). This interruption disrupts the direct flow of oxygenated blood from the heart to the lower body, necessitating collateral circulation through alternative vessels, such as the patent ductus arteriosus (PDA), to maintain perfusion, especially for lower extremity blood flow (6).

IAA is a rare condition, representing only 1% of congenital heart diseases, which is frequently associated with additional congenital cardiac anomalies, including ventricular septal defects and PDA, further complicating the clinical presentation (7). While the precise causes of IAA are still unclear, it is generally regarded as a phenotype arising from diverse aetiologies (8). For example, around 50%–80% of individuals with type B IAA have genetic origins, including DiGeorge syndrome and chromosome 22q11 deletion, with the deleted region being linked to cardiovascular development derived from the neural crest (8, 9).

Celoria and Patton have classified IAA into three types based on the site of aortic interruption concerning the arch vessels (10):

• Type A, where the interruption occurs at the isthmus between the left subclavian artery and the ductus;
• Type B, with the interruption in the distal AA between the left common carotid and left subclavian arteries;
• And type C, where the interruption is in the proximal AA between the innominate artery and the left common carotid artery (11).

Imaging is crucial for identifying these anomalies, significantly aiding in making accurate preoperative surgical decisions (12). AA anomalies in neonates and children can be identified using echocardiography and computed tomography (CT) (13).

Echocardiography alone can provide an accurate anatomical diagnosis of an IAA. It should identify the site of the interruption and measure the length of the arch discontinuity, the narrowest dimension of the left ventricular outflow tract, the diameter of the aortic annulus, and the ascending aorta. Additionally, it should define the location of any associated ventricular septal defect, assess the presence of the thymus (as its absence may indicate DiGeorge syndrome), and evaluate the presence and size of any associated atrial septal defect (11). Furthermore, Doppler ultrasonography can assess functional changes in vascular and surrounding structures (14).

Computed tomography angiography (CTA) serves as a valuable complementary diagnostic tool for unstable patients with IAA in urgent situations (15). The benefits of multislice CT, such as rapid scanning, which minimizes the need for sedation or general anesthesia, high spatial resolution, and the ability to assess both the airway and lungs simultaneously, have made it an effective method for evaluating a range of congenital heart diseases (16, 17). CT demonstrates 98% accuracy in diagnosing specific cardiovascular anomalies compared to a clinical consensus diagnosis, including surgical findings as the reference standard. Following an initial echocardiographic evaluation, CT could potentially replace diagnostic cardiac catheterization for detailed anatomical clarification in neonates (18).

Along with identifying the location of the interruption, both echocardiography and CTA should provide measurements of the length of the arch discontinuity, as well as the diameter of the ascending aorta (11). This is essential because an end-to-end (19) or end-to-side (20) anastomosis between the ascending and descending thoracic aorta is performed during a one-stage repair of IAA. Additionally, if patients have a severely hypoplastic ascending aorta (diameter <3 mm), homograft patch augmentation of the ascending aorta must be performed (21) or may lead to left ventricular outflow tract obstruction, which can significantly complicate patient management (22).

In cases of IAA, accurate delineation and identification of associated cardiac pathologies are crucial for effective preoperative decision-making (23). This study aims to perform a comparative analysis of echocardiography and CTA in assessing thoracic findings in patients with IAA.

## 2 Materials and methods

### 2.1 Patient population

This retrospective study included patients admitted for surgical treatment of IAA at the Heart Center of the University Medical Center in Astana, Kazakhstan, between September 2020 and January 2023. A total of 58 patients were included in the study, comprising 34 males (58.6%) and 24 females (41.4%), ranging in age from 1 to 821 days (median age: 18 days) (Table 1).

**Table 1:** Demographic profile of patients.

Characteristics	Options	Number
Sex (total number of patients—58)	Male	34 (58.6%)
	Female	24 (41.4%)
Age at surgery (days)	Max	821
	Min	1
	Median	18

Inclusion criteria were patients who had a preliminary diagnosis of aortic arch anomalies, with a high suspicion of IAA, based on echocardiographic evaluation performed at their local healthcare facilities. Only patients who were subsequently admitted to the Heart Center for further diagnostic work-up and surgical management, and who had complete clinical documentation and diagnostic imaging (echocardiography and CTA), were included in the study.

Exclusion criteria were the absence of complete clinical data or imaging studies, a final diagnosis that did not confirm IAA after comprehensive evaluation, or a history of prior surgical intervention involving the aortic arch.

Upon admission, all patients underwent further assessment at the radiology department, including echocardiography and CTA, to confirm the diagnosis and evaluate potential complications. Due to the observational nature of the study, no randomization or blinding was applied. All data, including clinical records and radiological images, were analyzed retrospectively.

Written informed consent was obtained from the legal representatives of pediatric patients for publication and any accompanying images. All procedures performed in studies involving human participants were in accordance with the ethical standards of the institutional and/or national research committee and with the 1964 Helsinki Declaration and its later amendments or comparable ethical standards. The study was approved by the Bioethics Committee of the Heart Center, University Medical Center, Astana, Kazakhstan (21 Jan 2022/No. 01-110/2022).

### 2.2 Imaging tools

Conventional echocardiographic measurements were performed by the Philips EPIQ 7 (Philips, Netherlands) ultrasound machine equipped with an S8-3 pediatric transducer (3–8 MHz). Two-dimensional, M-mode, and Doppler echocardiography were performed according to the American Society of Echocardiography recommendations (24).

CT investigations were performed using a Siemens Somatom Definition AS 64 multispiral CT scanner (Siemens, Germany) equipped with prospective ECG synchronization to minimize artifacts caused by cardiac motion. The patient was positioned supine for stable and standard imaging conditions. Intravenous administration of a nonionic, low-osmolality iodinated contrast material (Ultravist 370; Schering, Germany) was performed at a dose of 1.5–2.0 ml/kg, with a maximum volume of 120–150 ml, delivered via a dual-head pump injector (CT Motion XD8000; Ulrich Medical, Germany). The injection rate ranged from 0.5 to 2 ml/sec. Bolus tracking was utilized, with the marker placed in the ascending aorta and a start delay triggered at 100 Hounsfield units. Images were reconstructed with a slice thickness of 0.6 mm and a reconstruction increment of 0.1 mm. Multiplanar reformatted images were generated interactively on a dedicated workstation (Syngo VIA; Siemens, Germany).

### 2.3 Statistical analysis

Statistical analyses were performed using Stata version 18.0 (STATA, StataCorp, Texas, US). Data were assessed for univariate normality using the Shapiro–Wilk test. Continuous variables were presented as mean ± standard deviation (SD), medians, and interquartile ranges (IQR).

Categorical variables were expressed as counts and percentages, with comparisons conducted using the Chi-square or Fisher's exact test, as appropriate. Paired categorical data were analyzed utilizing McNemar's test. The Wilcoxon signed-rank test was used for paired data that were not normally distributed. Statistical significance was defined as *p* < 0.05 for all analyses.

Sensitivity and specificity were used to assess the accuracy of echocardiography and CTA, with 95% confidence intervals (CI) calculated using the Wilson score method.

## 3 Results

### 3.1 Accuracy in diagnosing IAA

Fifty-eight patients with IAA were evaluated using both echocardiography and CTA. The reference standard for diagnosis was established through clinical consensus, incorporating intraoperative findings where available, or a combination of imaging data, multidisciplinary evaluation, and clinical follow-up in nonsurgical cases.

Echocardiography correctly identified 53 out of 58 cases, missing 5 cases, resulting in a sensitivity of 91.4% (CI: 81.4%–96.3%) and a specificity of 100% (CI: 87.1%–100%). In comparison, CTA accurately diagnosed all 58 cases, achieving 100% sensitivity and 100% specificity.

To ensure the reliability of these results, all CTA findings were reviewed in the context of surgical outcomes or corroborated by consistent clinical and imaging follow-up when surgery was not performed. A diagnosis was considered a true positive if CTA findings were confirmed by operative observation or comprehensive clinical agreement. No instances of CTA-diagnosed IAA lacking subsequent confirmation were identified, supporting the observed 100% specificity.

McNemar's test was performed to compare the diagnostic accuracy of the two modalities. The results showed a statistically significant difference in diagnostic performance, with a *p*-value of <0.05, indicating that CTA outperforms echocardiography in detecting IAA. The results are summarized in Table 2.

**Table 2:** Diagnostic accuracy of echocardiography and CTA for interrupted aortic arch.

Modality	True positives (*n*)	False negatives (*n*)	Sensitivity (%)	Specificity (%)	*p*-value
Echocardiography	53	5	91.4%	100%	<0.05
CTA	58	0	100%	100%	<0.05

### 3.2 Measuring the diameter of the ascending aorta

The mean diameter of the ascending aorta measured by echocardiography was 0.95 cm (SD = 0.43 cm), with a median diameter of 0.875 cm and an interquartile range (IQR) of 0.7–0.96 cm. In comparison, the mean diameter measured by CTA was 0.89 cm (SD = 0.37 cm), with a median diameter of 0.8 cm and an IQR of 0.7–1 cm.

The Wilcoxon signed-rank test was used to evaluate the differences in aorta diameter measurements between echocardiography and CTA. Out of 58 paired measurements, 34 differences were positive, 23 were negative, and 1 was zero. The test statistic is z = 1.700, with an exact *p*-value of 0.09, indicating no statistically significant difference at the 5% level.

Overall, the Wilcoxon signed-rank test provided no sufficient evidence to conclude that there was a statistically significant difference between echocardiogram and CTA measurements of ascending aorta diameter.

### 3.3 Associated cardiovascular anomalies

Chi-square and Fisher's exact tests were applied to assess the clinical characteristics of patients with IAA and their associated chromosomal and cardiovascular anomalies, which were diagnosed using echocardiography and CTA. The results of these analyses are presented in Table 3.

**Table 3:** Clinical data of 58 patients with IAA and their associated cardiovascular anomalies.

Characteristics	Options	Number (Percentage)
Imaging modalities	64-slice CTA	58 (100.0%)
	Echocardiography	58 (100.0%)
Type of IAA	A	28 (48.3%)
	B	27 (46.6%)
	C	3 (5.2%)
Aortic arch side	Left	54 (93.1%)
	Right	4 (6.9%)
Chromosome abnormalities	DiGeorge syndrome	6 (10.3%)
	Down syndrome	1 (1.7%)
Combined cardiovascular anomalies	Ventricular septal defect	48 (82.8%)
	Atrial septal defect	34 (58.6%)
	Atrioventricular septal defect	3 (5.2%)
	Aortopulmonary window	4 (6.9%)
	Patent ductus arteriosus	58 (100.0%)
	Patent foramen ovale	34 (58.6%)
	Hypoplastic ascending aorta	20 (34.5%)
	Aberrant right subclavian artery	12 (20.7%)
	Truncus arteriosus	2 (3.4%)
	Anomalous pulmonary venous connection	2 (3.4%)
	Transposition of great arteries	8 (13.8%)
	Bicuspid aortic valve	17 (29.3%)
	Taussig-Bing anomaly	2 (3.4%)
Myocardial hypertrophy	Left	3 (5.2%)
	Right	3 (5.2%)
	Both	1 (1.7%)
Surgical treatment	One-stage total correction	56^a^ (96.6%)

^a^ Two patients died before corrective surgery.

## 4 Discussion

Our study compared the diagnostic accuracy of echocardiography and CTA for IAA.

IAA is a life-threatening condition with an absence of direct continuous blood supply to the lower body caused by a missing portion of the AA (25), which is accompanied by many cardiovascular anomalies, including PDA, ventricular and atrial septal defects, patent foramen ovale, hypoplastic ascending aorta, etc., as demonstrated in our study on Table 3. Each of these heart defects affects the course of the operation and the patient's further prognosis. Consequently, there is an increasing need for accurate radiological measurements and a comprehensive understanding of the lesion's anatomy during preoperative preparation (26).

The primary goal of surgical treatment for IAA is to restore normal blood flow by correcting the arch and associated cardiovascular defects. Prior to 2000, the standard surgical approach for IAA was staged repair. However, after 2001, it shifted to primary complete repair during the neonatal period (27), even with the complex intracardiac anomaly (28). This approach minimizes the need for multiple surgeries and improves long-term outcomes (29). Surgical intervention is essential for patient survival, as untreated IAA leads to a 90% mortality rate by a median age of 4 days (20).

The single-stage repair of IAA involves a direct anastomosis between the ascending and descending aorta after extensive mobilization. Since the procedure has become the approach of choice, the ascending aorta diameter is essential for surgical planning (30, 31), and many research findings emphasized the implication of ascending aorta measurements in cases of IAA. For instance, Schreiber et al. suggested that mobilizing the ascending aorta sufficiently would minimize tension around the anastomosis and reduce the risk of restenosis after one-stage primary correction (32). Furthermore, Kaulitz et al. revealed through echocardiographic measurements that the diameter of the ascending aorta was even related to the number of vessels originating from the proximal AA, indicating a connection to the type of IAA (33).

Among accompanying cardiovascular findings, hypoplasia of the ascending aorta is common in patients with IAA; in our sample, there are 34.5%. A more significant prevalence was reported by Vogel et al., who indicated, based on fetal echocardiographic data, a smaller-than-normal diameter of the ascending aorta in 73% of liveborn patients with IAA (34).

As for the measurement of the ascending aorta diameter, no statistically significant difference was observed between the echocardiography and CT in our sample. Blondheim et al. reported similar findings, showing a strong correlation between CT and echocardiographic measurements at various points along the ascending aorta, with the average measurement differences among their patients being clinically negligible (35). Similar results were revealed by the research of Tamborini et al., which concluded that in a cohort of patients with documented aortic dilatation or aneurysms, echocardiographic measurements of the proximal and ascending aorta demonstrated a strong correlation with CT measurements. These findings were characterized by minimal standard errors in the estimates and relatively low variability between and within observers (36).

**Figure 1:**
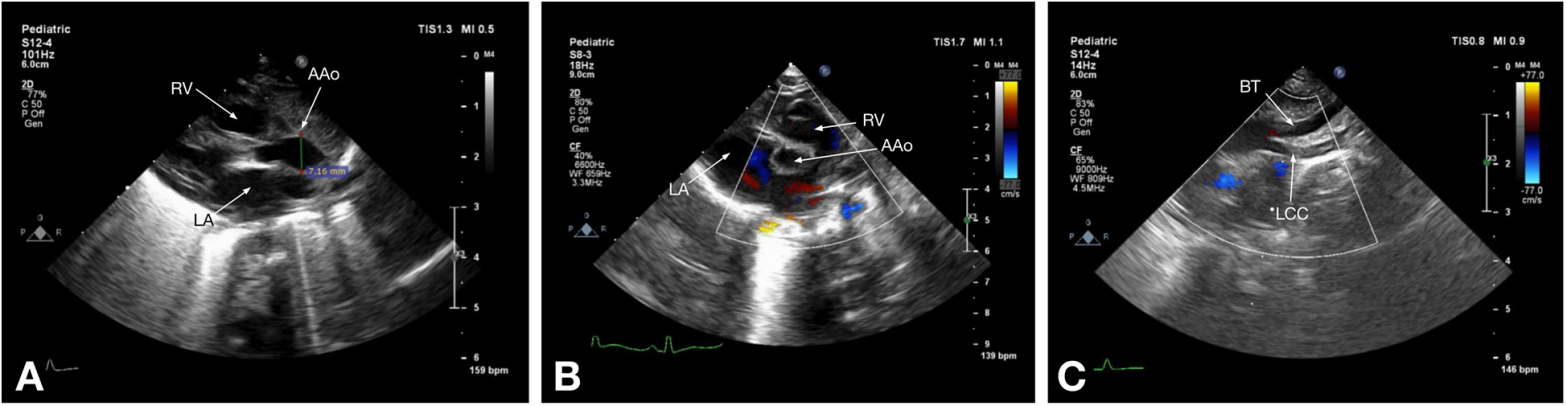
Echocardiographic images of a 7-day-old patient with type B IAA: **(A)** parasternal long-axis view shows the right ventricle (RV) and left atrium (LA) and the diameter of the ascending aorta (AAo) above the sinotubular junction; **(B)** parasternal short-axis view shows the right ventricle (RV), left atrium (LA), and the ascending aorta (AAo); **(C)** suprasternal notch view shows the brachiocephalic trunk (BT) and the left common carotid artery (LCC), followed by the echo signal disruption, corresponding to type B IAA.

Of the two imaging modalities used in our study, echocardiography is commonly employed as the initial imaging method, particularly for pediatric patients with IAA (Figure 1). However, since it is operator-dependent, it poses challenges when evaluating complex arch anomalies (12). In contrast, multislice CT offers high-resolution 3D imaging of IAA and collateral vessels. Combined with echocardiography, which detects intracardiac lesions, it typically provides enough information for preoperative planning in most cases (37). Recent advances in imaging modalities have improved accuracy and reliability (14), as evidenced by the lack of statistically significant differences between echocardiogram and CT measurements of ascending aorta diameter in our study.

In our study, CTA exceeded echocardiography in delineating IAA, exhibiting greater sensitivity and aligning closely with the findings of Al-Azzazy et al. (38). Their study reported a 100% sensitivity for CTA in diagnosing extracardiac aortic anomalies, surpassing the 92% sensitivity of Doppler echocardiography. Similar results were revealed by Soleimantabar et al., with the sensitivity and specificity of transthoracic echocardiography for detecting IAA at 75% and 100%, respectively, compared to CTA (39).

**Figure 2:**
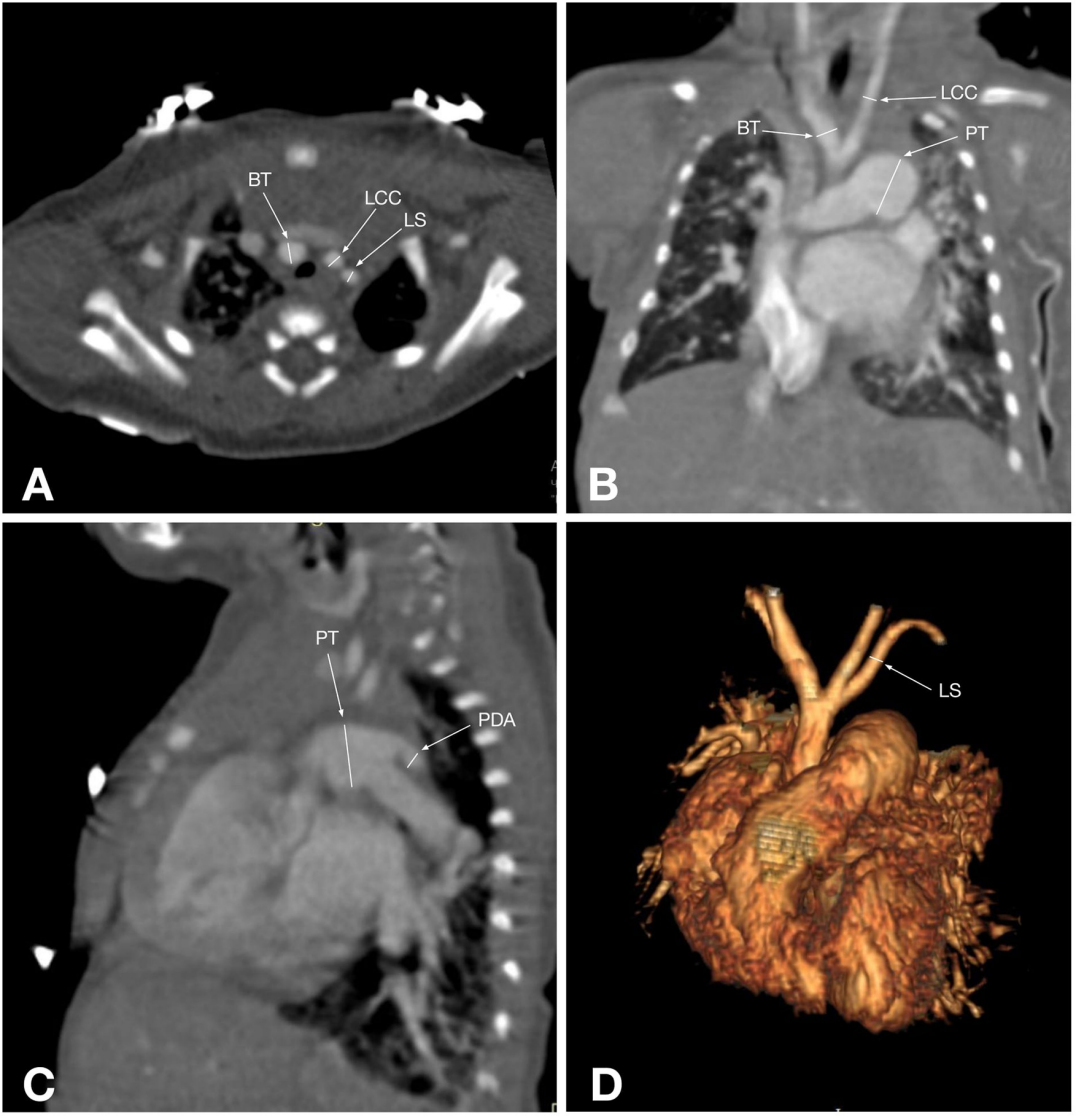
CT angiographic multiplanar images of a 2-day-old patient with type B IAA: **(A)** axial plane shows the ascending aorta (AAo), followed by the patent ductus arteriosus (PDA), which connects the dilated pulmonary trunk (PT) and the descending aorta (DAo); **(B)** coronal plane shows the ascending aorta (AAo) and its branch brachiocephalic trunk (BT), next to which are the superior vena cava (SVC) and the enlarged pulmonary trunk (red arrow); **(C)** Maximum intensity projection of the reformatted sagittal aorta image shows an interrupted aortic arch just distal to the left subclavian artery origin (LS) and the pulmonary trunk (PT), which is connected to the descending aorta via the patent ductus arteriosus (PDA); **(D)** 3D reconstruction shows an interrupted aortic arch after the right common carotid artery (RCC) arises, and the left subclavian artery (LS) originates from the descending aorta, corresponding to type B IAA.

**Figure 3:**
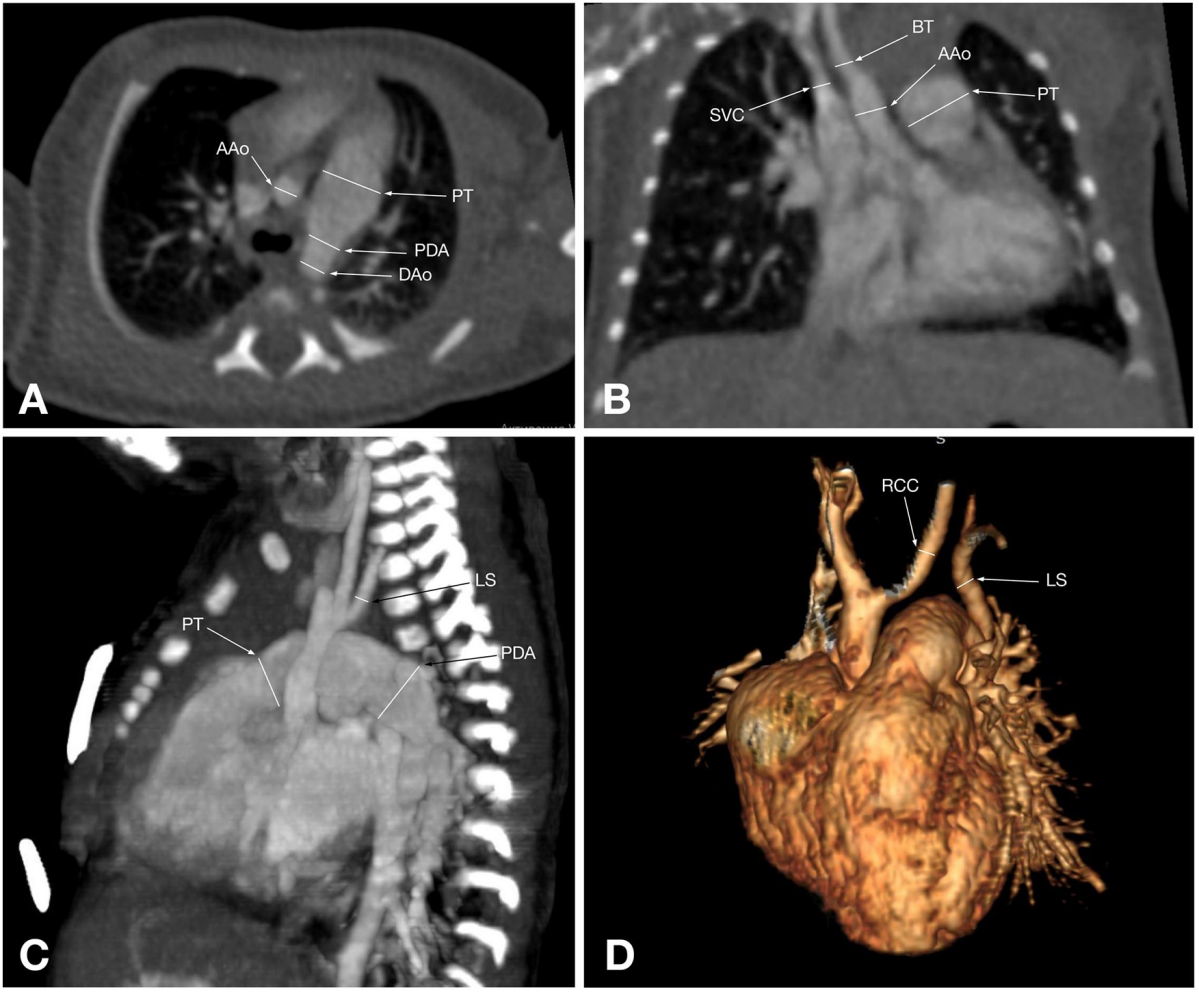
CT angiographic multiplanar images of a 55-day-old patient with type A IAA: **(A)** axial plane shows the branches of the aortic arch: brachiocephalic trunk (BT), left common carotid artery (LCC), and left subclavian artery (LS); **(B)** coronal plane shows that the ascending aorta branches into the brachiocephalic trunk (BT), which gives rise to the right common carotid artery. Also, the left common carotid artery (LCC) arising separately from the ascending aorta, the enlarged pulmonary trunk (PT), and its right branch are visualized; **(C)** Sagittal plane shows the pulmonary trunk (PT) continuing into the descending aorta via the patent ductus arteriosus (PDA); **(D)** 3D reconstruction shows an interrupted aortic arch after the left subclavian artery (LS) arises, corresponding to type A aortic arch interruption.

CTA provides a detailed visualization of AA anatomy and its spatial relationships with adjacent organs. Its advantage is further enhanced by various post-processing techniques, such as volume rendering, maximum intensity projection, and multiplanar reformatting, which can be applied to all structures within the scanned volume, making it superior to other imaging modalities for evaluating AA anomalies (5) (Figures 2, 3). Also, CTA is widely utilized for proper treatment and follow-up, as well as for preventing morbidities and mortalities (40). Traditionally, CT scans have been associated with high radiation exposure; however, recent advancements in radiation dose reduction technologies have significantly lowered the levels of exposure (41).

Table 4 compares echocardiography and CTA in diagnosing IAA, highlighting advantages and disadvantages. As demonstrated, echocardiography has multiple benefits, including availability and non-invasiveness, while CTA has disadvantages, such as radiation exposure and contrast-related issues. However, the critical advantage of CTA over echocardiography that makes it the method of choice is a comprehensive view of the AA and associated branches, including the precise location of the interruption, which is ideal for surgical planning.

**Table 4:** Comparative table of echocardiography and CTA.

Imaging modality	Advantages	Disadvantages
Echocardiography	Non-invasive Real-time imaging Portable and can be performed bedside Lower cost No radiation exposure	Operator-dependent Lower spatial resolution, especially for the distal aortic arch May miss subtle abnormalities Limited visualization due to overlying bony thorax
CTA	Higher resolution (slice thickness ≤0.6 mm), providing detailed visualization Provides a comprehensive view of the aortic arch and associated branches Precise location of the interruption Fast acquisition of high-resolution images (≈1–5 s for scan) Ideal for surgical planning	Radiation exposure (estimated effective dose: 1–4 mSv in neonates/infants) Need for contrast and patient preparation Requires a specialized CT machine in a clinical setting Higher cost due to advanced technology and contrast use

In addition to echocardiography and CTA, magnetic resonance imaging is a highly effective multiplanar imaging technique that allows AA malformations to be assessed without exposing patients to radiation. Both black blood and bright blood sequences, which do not require contrast, as well as contrast-enhanced magnetic resonance angiography, can be utilized and are synchronized with the cardiac cycle to minimize motion artifacts (42). It is also a practical option, but is more costly, time-consuming, and still more prone to artifacts compared to multislice CT (37).

While both imaging modalities, echocardiography and CTA, play crucial roles in diagnosis, treatment planning, and follow-up, CTA has demonstrated superior sensitivity and comprehensive anatomical detail. Understanding the complementary roles of these tools can optimize clinical outcomes by guiding accurate diagnosis and appropriate interventions.

This study has several limitations that should be acknowledged. First, its retrospective design limits control over confounding variables and introduces potential selection bias, which may affect the generalizability of the findings. Second, the relatively small sample size (*n* = 58) reduces the statistical power, particularly for subgroup analyses. For instance, the lack of a statistically significant difference in ascending aorta diameter measurements between echocardiography and CTA may be attributable to insufficient sample size, as suggested by the elevated *p*-value. Furthermore, not all patients underwent all imaging modalities, limiting the ability to perform direct modality comparisons, such as between CTA and other cross-sectional imaging modalities, such as magnetic resonance imaging. These constraints highlight the need for larger, prospective studies to further validate and expand upon the present findings.

## 5 Conclusion

These findings indicate that echocardiography is a practical initial diagnostic tool. Recent advances in medical imaging have enhanced its capabilities, making it comparable to CTA in measuring specific anatomical features, such as the diameter of the ascending aorta. However, CTA offers superior accuracy and should be prioritized when echocardiographic results are inconclusive or when detailed anatomical information is critical for diagnosis and surgical planning. Expanding future research to include additional imaging modalities and a larger sample size could significantly enhance our understanding of IAA visualization.

## Data Availability

The raw data supporting the conclusions of this article will be made available by the authors, without undue reservation.
